# Acute RNA Viral Encephalomyelitis and the Role of Antibodies in the Central Nervous System

**DOI:** 10.3390/v12090988

**Published:** 2020-09-05

**Authors:** Maggie L. Bartlett, Diane E. Griffin

**Affiliations:** W. Harry Feinstone Department of Molecular Microbiology and Immunology, Johns Hopkins Bloomberg School of Public Health, Baltimore, MD 21205, USA

**Keywords:** encephalomyelitis, antibodies, central nervous system

## Abstract

Acute RNA viral encephalomyelitis is a serious complication of numerous virus infections. Antibodies in the cerebral spinal fluid (CSF) are correlated to better outcomes, and there is substantive evidence of antibody secreting cells (ASCs) entering the central nervous system (CNS) and contributing to resolution of infection. Here, we review the RNA viruses known to cause acute viral encephalomyelitis with mechanisms of control that require antibody or ASCs. We compile the cytokines, chemokines, and surface receptors associated with ASC recruitment to the CNS after infection and compare known antibody-mediated mechanisms as well as potential noncytolytic mechanisms for virus control. These non-canonical functions of antibodies may be employed in the CNS to protect precious non-renewable neurons. Understanding the immune-specialized zone of the CNS is essential for the development of effective treatments for acute encephalomyelitis caused by RNA viruses.

## 1. Introduction

Encephalomyelitis (inflammation of the brain and spinal cord) is a dangerous presentation of numerous viral infections with potentially devastating long-term neurological sequelae. Virus-induced encephalomyelitis can be caused by primarily herpesviruses and RNA viruses including enteroviruses, rhabdoviruses, alphaviruses, flaviviruses, and bunyaviruses. In addition, many other viral families can cause acute encephalitis, such as paramyxoviruses and arenaviruses. However, this list is likely incomplete considering that most cases of viral encephalitis do not have an identified etiologic agent [1]. Despite the myriad of pathogens that cause viral encephalomyelitis, many features are shared. Foremost is the primary target cells, neurons, although some encephalitic viruses preferentially infect cerebrovascular endothelial cells or glial cells (Table 1) [2]. However, infection with most encephalomyelitis-causing viruses more often results in asymptomatic or mild febrile illness without neurologic disease. This is in part due to the rapidly mounted innate and adaptive immune responses to prevent the virus’s entry into the central nervous system (CNS). In addition, as this pathology can be caused by numerous viruses, there is considerable variability in mortality, ranging from <1 % for lymphocytic choriomeningitis virus (an arenavirus) to 100 % for rabies virus (a rhabdovirus) [3,4].

Moreover, pathogens induce diverse immune responses in the CNS despite its previous reputation as an immune-privileged zone. Today, it is more appropriately considered an immune-specialized zone in which many immune mechanisms exist, distinct from the rest of the immune system responsible for surveying primarily renewable cells. As this is not the case in the CNS, and damage would lead to long-lasting effects due to the irreplaceability of neurons, highly specialized responses have evolved to protect cells and clear infections. Despite viral encephalomyelitis being a prominent worldwide issue, traditional antiviral therapies fall short. To this end, a better understanding of the causation and resolution of viral encephalomyelitis may foster insight into new therapeutic approaches. While many studies of immune control have focused on innate mediators and microglia (a resident CNS cell), increasing evidence suggests an important role for antibody secreting cells (ASCs) [2,5,6,7]. One role of ASCs is certainly antibody production, which is critical in host defense against numerous pathogens. In addition to direct neutralization, antibodies drive clearance of viruses, bacteria, fungi, and parasites via interaction with the innate and adaptive immune systems. Antibodies can form complexes that sequester and allow for uptake of pathogens, clear toxins and infected cells, and increase antigen presentation, as well as regulating inflammation. Increasingly, we have seen evidence for a multitude of functions that ASCs and antibodies possess beyond the canonical functions [6]. Here, we review the known mechanisms by which ASCs and antibodies enter the CNS and contribute to the clearance of RNA viruses that infect neurons (Table 1).

### 1.1. Multiple Pathways for Viruses into the CNS

The most common CNS entry point is the blood, evidenced most by high viremia and replication in peripheral organs, which are correlated to the likelihood of CNS infection. However, the blood–brain barrier (BBB) inhibits direct access to the brain. To circumvent this protective tissue, some neurotropic viruses replicate in cerebrovascular endothelial cells, enter along with infected leukocytes, and cross directly to the cerebrospinal fluid (CSF), or in specialized cases by way of nerve terminals in peripheral organs or olfactory epithelium [28].

Viral encephalomyelitis typically presents with fever, headache, and evidence of neuronal dysfunction such as seizures, cognitive impairment, paralysis, and ataxia. These signs and symptoms of damage in the CNS are caused either directly by viral replication or often by the inflammatory response that ensues. The ability to overcome inflammation is thought to occur through the induction of regulatory T cells and suppression of lymphocyte function; however, recent studies have shed light on the important role B cells and antibodies play in CNS viral control as well as anti-inflammatory functions [2,6].

### 1.2. Recruitment of Antibody-Secreting Cells Predominant Over Antibodies Crossing the BBB

The brain was previously considered to be an immune privileged site lacking CNS surveillance by the immune system [29]. However, not only does the CNS contain its own assortment of antigen-presenting cells (astrocytes, microglia, endothelial cells, and pericytes), but CD4+ T cells enter in some frequency [30]. Although B cells were not commonly thought to enter the brain, increasing evidence has shown that they can enter the brain in healthy and infected individuals [31,32] and individuals infected with various pathogens [2,5,7,33]. The brain microenvironment changes in response to infection facilitate B cell entry into the CNS and support the proliferation, differentiation, and long-term survival of antiviral ASCs. For multiple encephalomyelitis-causing viruses, these changes include increased expression of common chemokines as well as concomitant infiltration of B cells expressing certain receptors (Table 2).

### 1.3. Antibodies Play Critical Roles in the Clearance of Many Acute Encephalomyelitis-Causing RNA Viruses

The focus of viral clearance from the brain has typically omitted B cells due to their low prevalence and the difficulty of crossing the BBB. However, it has long been established that intrathecal antibody in the CSF (indicating the presence of a local ASC) is predictive of the outcome and a hallmark of numerous human CNS infections, including the measles virus, rubella virus, poliovirus, varicella zoster virus, rubulavirus (mumps), herpes simplex virus, and Japanese encephalitis virus [33]. In the case of the measles virus, the presence of antibodies in the CSF is integral to the differential diagnosis of subacute sclerosing panencephalitis, a late disease due to persistent virus replication in the CNS [37]. Mounting evidence suggests that ASCs cross the BBB and are critical to the clearance of numerous encephalitic viruses, rather than antibodies alone entering from the periphery. A majority of what is known about this mechanism comes from studies of the Sindbis virus (SINV), rabies virus (RABV), West Nile virus (WNV), and murine hepatitis virus (MHV) in mice and is summarized here.

### 1.4. Alphaviruses

Alphaviruses are mosquito-borne single-stranded, positive-sense, enveloped RNA viruses that cause arthritis (Sindbis, Ross River, Semliki Forest, and chikungunya viruses) and encephalomyelitis (Venezuelan, western, and eastern equine encephalitis viruses). While the encephalitic alphaviruses are endemic to the Americas, the rapidly emerging arthritic alphaviruses that also cause neurologic disease can be found worldwide [38].

For the alphavirus family member SINV, ASCs enter the CNS to produce antiviral IgM and then IgG after infection [38]. Clinical studies on human viral encephalomyelitis suggest prompt IgM production was correlated to recovery, and recent studies on mice deficient in IgM, IgG, or both revealed that antibody was necessary and IgM or IgG was sufficient for clearance [38]. It is well characterized in SINV that both IFN-γ and antiviral antibody contribute to virus clearance from the CNS. Interestingly, despite working in concert, IFN-γ alone can clear the virus from spinal cord motor neurons, and antibody alone can clear the virus from the brain, as well as spinal cord neurons. Small numbers of IgM-secreting cells can be detected 3 days after infection, followed by IgG- as well as IgA-secreting cells by day 5 post infection [39,40,41]. Within 10–14 days, 80 % of the CNS B cells have class-switched and can be maintained for at least a year after recovery with enrichment for anti-SINV ASCs [39]. In vitro studies have shown that neither complement nor phagocytosis is needed for antibody-mediated SINV clearance, but bivalent activity of antibodies is required [42]. While there is a preponderance of evidence for ASCs and the role of antibody in the clearance of SINV from the CNS, not all mechanisms have been determined.

For the alphavirus SFV, antibody is required to suppress production of infectious virus, and B cells are present in the CNS and continue to produce antiviral antibodies for many months after infection [43,44]. However, viral RNA is not eliminated and remains capable of renewed replication if antibodies are no longer present. Unlike SINV, SFV infection in mice also results in CD8+ T-cell-mediated myelin loss [44] to which B cells may contribute by producing anti-myelin antibody [45].

### 1.5. Flaviviruses

The flaviviruses are arthropod-borne, single-stranded, positive-sense, enveloped RNA viruses that include the Zika virus, dengue virus, and West Nile virus (WNV). One of these, WNV, is the leading cause of mosquito-borne disease in the United States. While 80% of human infections are asymptomatic, up to 50 % of patients that develop encephalomyelitis have long-term neurological sequelae [46]. An early antibody response has been demonstrated to be important in containing viremia and limiting dissemination of WNV to the CNS. Similar to SINV, both IgM and IgG are protective in the CNS for WNV [47]. Furthermore, lack of B and T cells, but not T cells alone, increases mortality in mice after infection with WNV in the CNS [48]. It is hypothesized that anti-WNV IgM induces a mature humoral response by complement activation and facilitation of production of T cell-dependent and T cell-independent antibody [49].

### 1.6. Coronaviruses

Coronaviruses are single-stranded, positive-sense, enveloped RNA viruses that include common cold coronaviruses as well as three pandemic viruses: Severe Acute Respiratory Syndrome virus (SARS), Middle East Respiratory Syndrome virus (MERS), and SARS-Coronavirus 2 (SARS-CoV-2). The neurologic manifestations of SARS-CoV-2 span encephalomyelitis, anosmia, and Guillain–Barre syndrome [50]; however, little is known about the neurotropism or mechanisms of CNS clearance. Interestingly, anti-SARS-CoV-2 antibodies are detected in the CSF [51], suggesting potential entry of antibodies or ASCs to the CNS. While much is being learned quickly during the current pandemic, significant work has already been done to understand CNS clearance mechanisms in mice using the mouse coronavirus, murine hepatitis virus (MHV). While MHV is primarily controlled by CD8+ T cells, B cells make significant contributions, particularly in preventing virus recrudescence [52]. Studies on MHV strain A59 revealed that mice deficient in antibody production could not clear MHV from the CNS [53]. In the case of MHV strain JHM mouse hepatitis virus (JHMV), many of the chemokines, cytokines, and surface markers are similar to those expressed during SINV infection and support the concept of a shared process for ASC recruitment and accumulation in the CNS (Table 2) [54].

### 1.7. Rhabdoviruses

Rhabdoviruses are single-stranded, negative-sense, enveloped RNA viruses that include the rabies virus (RABV), which has been recognized as a fatal human disease since the times of the ancient Egyptians and Mesopotamians [55]. While T cell activity is associated with RABV replication control and long-term survival, B cells are critical to viral elimination. Further, this is primarily due to RABV-specific antibodies produced by the infiltrating B cells rather than antibodies crossing the BBB from the periphery [56]. Moreover, non-lethal cases of RABV in dogs have been linked to the presence of anti-RABV antibodies in the CSF due to a leaky BBB [57]. This phenomenon has also been well characterized in mice, in which CNS inflammation disrupts the BBB so that RABV-specific immune effectors can infiltrate and clear the virus [58]. Despite the apparent recovery of experimentally infected animals, the few human survivors have long-lasting sequelae, suggesting permanent damage [58]. To this end, the residency of ASCs and their contributions to long lasting pathology or control of recrudescence are of interest but difficult to address, considering the high lethality and inability to safely study the disease in humans during the disease’s progression.

### 1.8. Clearance of Virus from the CNS without Antibody

**Figure 1:**
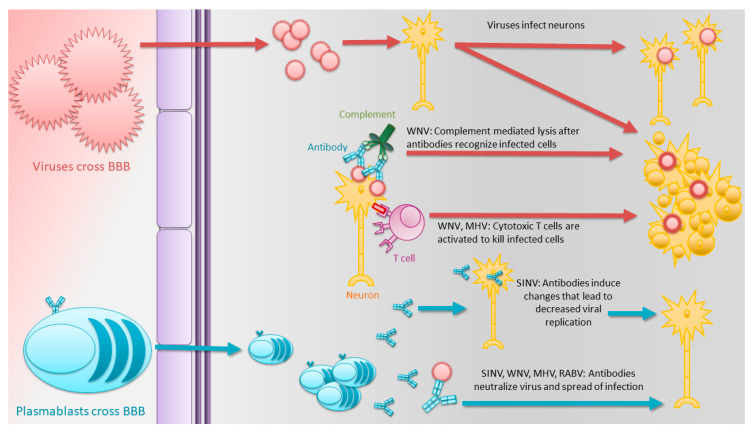
Antibody-mediated viral clearance from the central nervous system (CNS). Viruses cross the blood–brain barrier (BBB) to infect neurons. Without control, the virus continues to spread and infect cells in new regions of the CNS. The ensuing innate immune response attracts plasmablasts produced in secondary lymphoid tissue to cross the BBB and produce antibodies in the CNS. Multiple mechanisms in addition to neutralization are involved in antibody-mediated control and clearance of the infectious virus from neurons.

Viral clearance is a complex multi-step process that does not conform to a one-size-fits-all model and may be dependent on the type of CNS cell infected. Another flavivirus, the Zika virus (ZIKV), infects the CNS, but clearance is dominated by resident microglia and infiltrating monocytes/macrophages and does not require B and T cells [59]. Studies of the alphavirus Venezuelan equine encephalitis virus (VEEV) suggest that while antibody responses are important, B-cell-deficient mice can recover, suggesting a role for T cells in clearance [60]. However, while not all encephalomyelitis-causing viruses require antibody or ASCs for clearance, many do. Further, perhaps more fascinating is the variation in how antibodies are utilized depending on etiologic agent (Figure 1).

### 1.9. Non-Canonical Functions of Antibodies in the CNS and Potential Future Avenues of Study

While studies to date have focused on the ability of ASCs or antibody to mediate viral clearance in the CNS by neutralization, other antibody-mediated mechanisms are likely to be of importance. Given that WNV in part relies on complement-mediated lysis for clearance (in which the complement binds to the Fc portion of the antigen-bound antibodies, leading to activation of the classical pathway), there may be cases of encephalomyelitis that are overcome by other common features of antibodies. These include antibody-dependent cellular cytotoxicity (in which antibody-bound infected cells are killed by NK cells), antibody-dependent phagocytosis (in which antibody-bound infected cells are phagocytosed), the release of cytotoxic molecules, degranulation, or via immune regulation. As these methods typically result in cell death, their contributions to clearance in the CNS and recovery from infection are likely minimal considering the irreplaceability of neurons. Thus, attractive possible functions of antibodies in the CNS beyond neutralization are mechanisms of clearance that do not require cell lysis. Beyond the typical functions associated with antibodies, more atypical actions have been described.

### 1.10. Antibody Synergism

The B cell response is complex and polyclonal and thus the antibody response has been well documented to act synergistically to neutralize viruses [61,62,63,64,65]. One mechanism for synergy is the binding of one antibody to a virus to induce a conformational change that exposes a second site for a different antibody to bind and neutralize. This has been documented for the Ebola [66], herpes simplex [67], and tick-borne encephalitis [68] viruses. This mechanism is widely used in the periphery, but the extent to which antibody synergism is used in the CNS is unclear, as many studies focus on the use of monoclonal antibodies for which individual antibody neutralization efficacy is already known.

### 1.11. Modifications and Auxiliary Features of Fc

Another feature that may aid in viral neutralization within the CNS are Fc modifications. One such modification, sialylation, is mechanistically considered to be anti-inflammatory but may also have direct antiviral effects [69,70,71,72,73,74,75]. For instance, sialic acid on secretory IgA can aid in neutralization of the influenza virus by binding to hemagglutinin for competition with binding to the cell surface sialic acid needed for entry [76]. Additionally, many modifications on Fc domains in the periphery correlate to patient outcomes of viral diseases [77] but have not been examined extensively for antibodies within the CNS.

### 1.12. Antibodies with Direct Microbe Killing or Proteolytic Activity

While antibodies typically kill pathogens via neutralization, complement-mediated lysis, or FcR-dependent phagocytosis, there is growing evidence that antibodies themselves can induce microbe killing [78,79,80]. Binding by antibodies to a bacterial surface can induce gene expression changes that interfere with bacterial quorum sensing, resulting in pathogen clearance [76]. New evidence suggests that antibodies can have proteolytic activity to hydrolyze a pathogen-specific protein, which can compromise the viral life cycle. This activity has been documented for IgM, IgG, and IgA from healthy individuals against the HIV-1 envelope [81,82] as well as for the influenza virus [83]. In addition, this activity is well documented in numerous autoimmune and neurodegenerative conditions, although the mechanism and antibody contributions under these conditions are still being elucidated [83]. Intriguingly, of the antibodies with known crystal structures, 7 % have a serine protease catalytic triad that could potentially have proteolytic activity [83]. This noncytolytic mechanism inhibits viral propagation by the destruction of key viral proteins and could be useful in clearance of the virus from the CNS without loss of neurons.

### 1.13. Antibodies Able to Suppress Virus Replication Intracellularly

An interesting feature of antibodies to the E2 glycoprotein of SINV is the ability of bivalent antibody to modulate virus replication intracellularly. Antibody treatment leads to decreased viral transcription/translation, inhibition of viral budding, and restoration of host transcription/translation in both SINV-infected mice and cell culture [84,85]. Further studies have highlighted that antibody treatment leads to decreases in SINV subgenomic RNA in neuronal cells [86]. Antibodies can also be internalized with a viral target, allowing for intracellular receptor binding, neutralization via the proteasome, and stimulation of the NF-κB pathway, as demonstrated for adenovirus and tripartite motif-containing 21 (TRIM21) [87]. Modulation of intracellular viral replication could be a potent mechanism of control by antibodies, particularly in precious non-renewable cells such as neurons.

## 2. Conclusions

Acute encephalomyelitis caused by RNA viruses can be fatal, result in perpetual disability due to permanent damage to neurons, or be controlled by the immune response followed by recovery. For many viral infections of the CNS, the prompt appearance of antiviral antibodies produced by ASCs within the CNS is associated with recovery. However, noncytolytic virus clearance may result in the persistence of nucleic acid in the CNS and the need for long-term CNS-resident immune cells to prevent recrudescence or progressive disease. The antibody response and its protective role in numerous viral encephalopathies have been documented but mechanisms are less well defined. However, numerous noncytolytic antibody functions have been identified and could be critical to the control of viral infections in non-renewable cells like neurons. Understanding this facet of CNS immunity is imperative to understanding this immune-specialized zone, as well as being necessary for the development of therapeutics to prevent neurologic disease.

## Figures and Tables

**Table 1:** Select causes of acute viral encephalomyelitis in animal models and humans.

Virus	Viral Family	Target Location	Target Cells	Animal Species	Additional Human CNS Presentation	Reference
Eastern equine encephalitis	*Togaviridae*	Olfactory bulb, widespread	Neurons	Mouse	None associated	[8,9]
Western equine encephalitis	*Togaviridae*	Olfactory bulb, substantia nigra widespread	Neurons	Mouse	Similar to Parkinson’s, cogwheel rigidity	[10]
Venezuelan Equine Encephalitis Virus	*Togaviridae*	Olfactory bulb, widespread	Neurons	Mouse	None associated	[11,12]
Sindbis virus	*Togaviridae*	Hippocampus and brainstem	Immature and mature neurons	Mouse	None associated	[13,14]
Semliki Forest Virus	*Togaviridae*	Corpus callosum	Neurons, oligodendrocytes	Mouse	None associated	[15,16]
Chikungunya virus	*Togaviridae*	Not determined	Astrocytes	Mouse, human cell culture	Guillain–Barre syndrome	[17]
Japanese encephalitis virus	*Flaviviridae*	Basal ganglia	Neurons	Rat	Similar to Parkinson’s	[18]
Zika virus	*Flaviviridae*	Frontal cortex, hippocampus, striatum	Mature neurons	Mouse, human cell culture	Memory impairment	[19]
Severe acute respiratory syndrome coronavirus 2	*Coronaviridae*	Not determined	Presumed olfactory neurons	Human	Guillain–Barre syndrome, smell/taste dysfunction	[20,21]
JHM mouse hepatitis virus	*Coronaviridae*	Not determined	Neurons, Glia cells	Mouse	None associated	[22,23]
Poliovirus	*Picornaviridae*	Brainstem/spinal cord	Motor neurons	Mouse	Paralysis	[24]
Theiler’s murine encephalomyelitis virus	*Picornaviridae*	hippocampus periventricular thalamic nuclei; septal nuclei; and piriform, parietal, and entorhinal cortices	Glia, macrophages	Mouse	spontaneous recurrent epileptic seizures	[25]
Nipah virus	*Paramyxoviridae*	Cribriform plate, olfactory bulb	Neurons	Hamster	None associated	[26]
Rabies virus	*Rhabdoviridae*	Not determined	Neurons	Mouse	Agitation, cognitive dysfunction	[27]

Table of select viruses known to induce encephalomyelitis including known target cells, regions, and unique presentations associated with infection. General meningoencephalomyelitis symptoms include but are not limited to: headache, light sensitivity, neck stiffness, lethargy, increased irritability, seizures, skin rashes, difficulty talking or speech changes, changes to alertness, confusion, hallucinations, loss of energy, loss of appetite, unsteady gait, nausea and vomiting, loss of muscle power in extremities, double vision, hearing/speech impairment, coma.

**Table 2:** Chemokines, cytokines, and surface receptors associated with antibody-secreting cell (ASC) recruitment and maintenance in the central nervous system (CNS).

Virus	Abbr.	Chemokines	Cytokines	Surface Receptors	Reference
Sindbis virus	SINV	CXCL9, CXCL10, CCL1, CCL2, CCL5	BAFF, IL−10, and IL−21	CXCR3, CXCR5, CCR7	[2]
West Nile virus	WNV	CXCL9, CXCL10, CCL2, CCL5, CCL7	TNF-α, IFN-γ	CXCR3, CCR1, CCR2, CCR5	[34]
JHM mouse hepatitis virus	JHMV	CXCL9, CXCL10	APRIL, BAFF, IL−6, IL−10, IL−21	CXCR3, B220, sIg, CD19	[7,33]
Rabies virus	RABV	CXCL10, CX3CL1, CCL4, CCL5, CCL7, CCL21	IL−6, IL−1, IL−12, TNF-α, IFN-γ	Unknown	[35,36]
